# Lung cancer specialist physicians’ attitudes towards e-cigarettes: A nationwide survey

**DOI:** 10.1371/journal.pone.0172568

**Published:** 2017-02-24

**Authors:** Dong Wook Shin, Young Il Kim, Seung Joon Kim, Jung Soo Kim, SeMin Chong, Young Sik Park, Sang-Yun Song, Jin Han Lee, Hee Kyung Ahn, Eun Young Kim, Sei Hoon Yang, Myoung Kyu Lee, Deog Gon Cho, Tae Won Jang, Ji Woong Son, Jeong-Seon Ryu, Moon-June Cho

**Affiliations:** 1 Department of Family Medicine & Health Promotion Center, Seoul National University Hospital, Seoul, Korea; 2 Cancer Survivorship Clinic, Seoul National University Cancer Hospital, Seoul, Korea; 3 Department of Radiation Oncology, Chungnam National University School of Medicine, Daejeon, Korea; 4 Department of Internal Medicine, Seoul St. Mary Hospital, The Catholic University of Korea College of Medicine, Seoul, Korea; 5 Department of Internal Medicine, lnha University Hospital, Inha University College of Medicine, Incheon, Korea; 6 Department of Radiology, Chung-Ang University Medical Center, Chung-Ang University College of Medicine, Seoul, Korea; 7 Department of Internal Medicine, Seoul National University Hospital, Seoul, Korea; 8 Department of Thoracic and Cardiovascular Surgery, Chonnam National University Hwasun Hospital, Chonnam National University Medical School, Hwasun, Korea; 9 Medical Correspondent & Social Policy Desk, Donga-A Ilbo, Seoul, Korea; 10 Department of Internal Medicine, Gachon University Gil Medical Center, Incheon, Korea; 11 Department of Internal Medicine, Wonkwang University College of Medicine, Iksan, Korea; 12 Department of Internal Medicine, Wonju College of Medicine, Yonsei University, Wonju, Korea; 13 Department of Thoracic and Cardiovascular Surgery, St. Vincent's Hospital, College of Medicine, The Catholic University of Korea, Suwon, Korea; 14 Department of Internal Medicine, Kosin University Medical College, Pusan, Korea; 15 Department of Internal Medicine, Konyang University Hospital, Daejeon, Korea; Istituto Di Ricerche Farmacologiche Mario Negri, ITALY

## Abstract

**Objectives:**

Despite a sharp increase in e-cigarette use, there is debate about whether e-cigarettes are a viable alternative for harm reduction, and the forms that regulation should take. Healthcare providers can be effective in offering guidance to patients and their families and shaping regulatory policy. We described lung cancer specialists’ attitudes toward e-cigarettes and its regulation.

**Methods:**

We undertook a nationwide survey of pulmonologists, thoracic surgeons, medical and radiological oncologists who are members of Korean Association for Lung Cancer. Survey items included beliefs and attitudes toward e-cigarettes, attitudes toward e-cigarette regulation and preparedness on discussing e-cigarettes with their patients.

**Results:**

Most respondents believed that e-cigarettes are not safer than conventional tobacco cigarettes (75.7%) or smokeless tobacco (83.2%), and feared that discussing e-cigarettes with the patients would encourage use (65.4%). They did not consider it a smoking cessation treatment (78.3%), and thus would not recommend it to smokers who do not want to quit (82.2%) or who failed to quit with conventional smoking cessation treatment (74.1%). Most respondents supported all examples of e-cigarette regulations, including the safety and quality check (97.8%), warning label (97.8%), advertisement ban (95.1%), restriction of flavoring (78.4%), minimum purchasing age (99.5%), and restriction of indoor use (94.6%). Most learned about e-cigarettes from media and advertisements, or conversation with patients rather than through professional scientific resources, and reported discomfort when discussing e-cigarette with patients.

**Conclusion:**

Lung cancer specialist physicians in Korea doubt the safety of e-cigarette and use of e-cigarette as smoking cessation treatment, and supported strict regulation. However, only 20% reported that they obtained information on e-cigarettes from the scientific literature and many lacked adequate knowledge based on scientific evidence, suggesting the need for better preparedness. Nevertheless, the views of professionals revealed from our study could help to develop clinical guidelines and regulatory guidance.

## Introduction

With stronger tobacco control, such as clean indoor air laws and cigarette tax increase, public interest in e-cigarettes has increased rapidly [1]. E-cigarette use has increased sharply in the US [2] and UK [3]. In Korea, prevalence of ever and current e-cigarette use was 6.6 and 1.1% respectively in 2013 [4]. Dual use of e-cigarette with conventional tobacco cigarettes is increasingly common [5].

E-cigarettes are actively promoted through media channels, sports or music events, and outdoor or in-store displays [6]. They are usually portrayed as a safer alternative to conventional tobacco cigarettes or a smoking cessation aid [7], and perceived as such by smokers themselves. [3]. Smokers who ever used e-cigarettes reported less desire to quit smoking, reduction or cessation of conventional tobacco cigarettes, improved subjective health status, and recommended e-cigarettes to other smokers [8].

There is debate about whether e-cigarettes are a viable alternative for harm reduction. Opponents urge caution, warning of potential toxicity, questioning quality standards in manufacture, adequacy and consistency in nicotine delivery, and long-term effect of propylene glycol inhalation [9]. Proponents claim that e-cigarettes, in theory, are less harmful than conventional tobacco cigarettes because they do not produce the same dangerous combustion by-products [10]. The World Health Organization [11], World Lung Foundation [12], and World Medical Association warn against e-cigarette use. However, British Medical Association [13], Public Health England [14], and American Association of Public Health Physicians [15] admit a role of e-cigarette in potential harm reduction.

With limited data to guide e-cigarette regulation, in many countries, e-cigarettes are freely marketed, often with the claim that they aid smoking cessation, and e-cigarettes use is generally allowed in home, in the public transportation, and at work [8]. Evidence is mixed regarding the effectiveness of e-cigarettes as a supporting cessation aid [16–19], the health effects of secondhand exposure to vapors from e-cigarette [20] and e-cigarette’s role as the gateway to conventional tobacco cigarette smoking [21].

There are differing perspectives regarding whether and how e-cigarettes should be regulated [8,22,23]. In 2016, the US Food and Drug Administration (FDA) finalized a rule to expand its tobacco regulatory authority to e-cigarettes, and now regulates the manufacture, import, packaging, labeling, advertising, promotion, sales, and distribution of e-cigarettes, including product registration with FDA, warning label, and minimum age of sale [24]. The UK government allows prescription of e-cigarettes for patients trying to quit smoking [25]. In Korea, e-cigarette with nicotine is regulated as a tobacco product by Ministry of Strategy and Finance, and Ministry of Health and Welfare, and e-cigarette without nicotine is regulated by Ministry of Food and Drug Safety (equivalent to FDA in the US). As of 2015, the minimum age for purchase is 19, and e-cigarette use is prohibited in enclosed public spaces and on public transportation. The Korean government applies a special health tax to e-cigarettes proportional to USD 1.65 per mL nicotine liquid, and there are some restrictions on e-cigarette advertising, promotion or sponsorship.

E-cigarette use is increasing among patients with cancer. Although a group of such patients were strongly advised to quit smoking at the time of diagnosis and during cancer treatment, some of them had high nicotine dependence, failed to quit smoking despite several attempts, and finally chose e-cigarette use [26]. A US study found that 38.5% of cancer patients who were enrolled in a tobacco treatment program at a comprehensive cancer center were using e-cigarettes, a sharp increase in recent years [26].

Healthcare providers are crucial in prevention by giving guidance to patients and their families about risk behaviors, including tobacco use [22]. Lung cancer specialist physicians have great potential to educate their patients and family members about e-cigarettes. In the current situation with limited evidence, the relevant health professionals’ opinions could be important information in shaping regulatory policy while waiting for further evidence. Therefore, we surveyed their 1) beliefs and attitudes toward e-cigarettes, 2) attitudes toward e-cigarette regulation and 3) preparedness on discussing e-cigarettes with their patients, using a nationally representative sample.

## Methods

### Study design and subjects

The present study is part of a nationwide survey to explore the views of lung cancer specialists regarding smoking-related policies in Korea. This web-based survey was conducted in October 2015. The study was approved by the Institutional Review Board of the Inha University Hospital, Incheon, Korea (IRB no. 15–053).

Potentially eligible subjects were physicians who are members of the Korean Association for Lung Cancer, a representative multidisciplinary physician society in Korea. The society is open to physicians of all disciplines, but we included only pulmonologist, thoracic surgeon, medical oncologist, and radiological oncologist in this study, as these specialists are in direct contact with lung cancer patients, so the survey contents are most pertinent. Three hundred and eighty-three physicians were eligible, and were invited to participate in the study.

Eligible lung cancer specialists were sent up to three invitations per unique email address to participate in the study with a message to remind them of the survey participation. For non-responders, one phone call was made to remind them of the survey. Informed consents were obtained through the cover letter of the e-mail survey, and survey responses were confidential and recorded in an encrypted database. No incentive was offered for survey completion.

Of 383 eligible physicians, 196 agreed to participate in the study (51.3% participation rate). However, three did not provide sufficient responses to the e-cigarette questionnaire and were excluded from the analysis, and eight responded that they are not aware of e-cigarettes, leading to a final sample of 185 (48.3% effective response rate).

### Measures

We developed e-cigarette questionnaire items based on previous research with similar topics S1 File [22,27]. The questionnaire included questions regarding lung cancer specialist physicians’ beliefs and attitudes toward e-cigarettes, attitudes toward e-cigarette regulation, and preparedness for counseling about e-cigarettes. Twelve lung cancer specialist clinicians and one survey specialist reviewed the survey measures to identify potential sources of response error and improve survey items, and a pilot test was performed.

#### Beliefs and attitudes toward e-cigarette

Risk beliefs about e-cigarettes were assessed by asking whether they believe e-cigarettes are safer than conventional tobacco cigarettes or smokeless tobacco (chew, snuff, dip), and whether they believe that it could be a gateway to other tobacco products. These questions were used in a US study which examined healthcare providers’ beliefs and attitudes toward e-cigarette use for adolescent patients [22]. Attitudes toward communication about e-cigarettes were asked with questions adopted from the same study, and included whether they believe discussing e-cigarettes with patients could encourage them to use e-cigarettes, whether they believe it important to discuss e-cigarettes with the patients and whether smokers need to know about e-cigarettes [22].

#### Attitudes toward e-cigarette regulations

Participants were asked to what extent they agreed (4-point Likert scale questions, strongly disagree to strongly agree) with advertisement ban, warning label, regulation by FDA, flavoring ban, indoor smoking ban, and minimum legal age of purchase [8,27].

#### Preparedness for e-cigarette use counseling

The respondents were asked about their sources of information (e.g. professional source, media or advertisement, and conversation with the patients) [22]. They were asked whether they had ever cared for patients who used e-cigarette, and their comfort level with discussing e-cigarette with their patients (with 4-point Likert scale, very uncomfortable to very comfortable). For comparison purposes, they were asked about their comfort level with discussing smoking cessation treatment [28].

#### Demographic and professional characteristics

The survey inquired about age, gender, specialty, years since board certification, and patient volume (clinical practice time per week, average number of overall and lung cancer outpatients per week). Physicians’ own smoking practice (current vs. past vs. none) was asked. Characteristics of the physician’s workplace were asked (university hospital vs. specialized cancer center, private vs. public, and geographic location).

### Statistical analysis

Descriptive statistics were calculated for all questions. The attitude responses by smoking status of the lung cancer physicians (never smokers vs. ever smokers) were compared with chi-square tests. Multivariate logistic analyses were performed to investigate predictors of each attitude after dichotomization (strongly agree, agree vs. disagree, strongly disagree) with a age (<45 and ≥45 years), sex, and smoking status as independent variables. All statistical analyses were conducted using STATA version 14.0 (STATA Corp, College Station, TX, USA). Null hypotheses of no difference were rejected if p-values were less than .05, or, equivalently, if the 95% CIs of risk point estimates excluded 1.

## Results

### Respondent characteristics

Most providers were male (81.4%) and practiced in a university hospital or cancer center hospital setting (83.4%). Respondents included pulmonologists (56.8%), thoracic surgeons (23.2%), radiation oncologists (10.3%) and medical oncologists (9.7%). The average age was 44.5 years (SD 7.2 years), and the mean time from board certification was 13.8 years (SD 7.2 years) (Table 1).

**Table 1:** Baseline Characteristics of Participants (n = 185).

	N	%
Age (mean, SD)	44.5	7.2
Gender		
Male	149	80.5
Female	36	19.5
Smoking status		
Current smoker	9	4.9
Past smoker	67	36.2
Non-smoker	93	50.3
Missing	16	8.7
Specialties		
Pulmonologist	105	56.8
Thoracic surgeon	43	23.2
Medical oncologist	18	9.7
Radiation oncologist	19	10.3
Years from board certification (mean, SD)	13.8	7.2
Hospital type		
University hospital	142	76.8
Cancer specialty hospital	12	6.5
Secondary hospital	13	70
No answer	18	9.7
Hospital type		
Public hospital	46	24.9
Private hospital	121	65.4
No answer	18	9.7
Number of clinical sessions per week (mean, SD)	4.1	1.4
Number of lung cancer patients per week (mean, SD)	32.5	41.0

### Beliefs and attitudes toward e-cigarettes

Most respondents disagreed that e-cigarettes are safer than conventional tobacco cigarettes (75.7%) or smokeless tobacco (83.2%), and they expressed considerable concern that e-cigarettes could be a gateway to other tobacco use (83.8%). The majority believe that smokers need to know about e-cigarette (81.1%), and it is important to discuss e-cigarettes with the patients (67.6%), but at the same time, they worried that discussing e-cigarettes with patients may encourage use (65.4%). Most respondents disapproved of e-cigarettes as a smoking cessation treatment (78.3%), and would not recommend e-cigarettes to smokers who do not want to quit (82.2%), or smokers who failed to quit with conventional smoking cessation treatment (74.1%) (Table 2). There was no significant difference in attitudes toward e-cigarettes by smoking status of the lung cancer specialist physicians, and no significant predictors were found in multivariate logistic analyses (data not shown).

**Table 2:** Attitudes towards E-Cigarettes among Participants.

	Strongly disagree		Disagree		Agree		Strongly agree	
	N	%	n	%	n	%	n	%
E-cigarette risk beliefs								
E-cigarettes are safer to use than conventional tobacco cigarettes	42	22.8	98	53.1	41	22.2	4	2.2
E-cigarettes are safer to use than smokeless tobacco	34	18.4	120	64.9	28	15.1	3	1.6
E-cigarettes could be a ‘gateway’ to other tobacco use	5	2.7	25	13.5	119	64.3	36	19.5
Communication								
Discussing e-cigarettes with patients may encourage them to use e-cigarettes	4	2.2	60	32.4	100	54.1	21	11.4
It is important to discuss e-cigarettes with the patients	6	3.2	54	29.2	101	54.6	24	13.0
Smokers need to know about e-cigarettes	5	2.7	30	16.2	116	62.7	34	18.4
E-cigarette for smoking cessation								
E-cigarettes can be regarded as a type of smoking cessation treatment.	40	21.6	105	56.8	37	20.0	3	1.6
It is better to recommend e-cigarette to smokers who do not want to quit.	43	23.2	109	58.9	31	16.8	2	1.1
It is better to recommend e-cigarette to smokers who failed to quit with conventional smoking cessation treatment	37	20.0	100	54.1	45	24.3	3	1.6

### Attitudes toward e-cigarette regulations

The large majority of respondents agreed that e-cigarette advertising should be banned (95.1%), e-cigarettes should be regulated by the FDA for safety and quality (97.8%), should carry warning labels about their potential risks (97.8%), use of e-cigarettes indoors should not be allowed (94.6%), and there should be a minimum legal age to purchase e-cigarettes (99.5%). The majority (78.4%) answered that the sale of fruit or candy-flavored e-cigarettes should be banned (Table 3).

**Table 3:** Attitudes towards E-Cigarette Regulation among Participants.

	Strongly disagree		Disagree		Agree		Strongly agree	
	N	%	n	%	n	%	n	%
E-cigarette advertising should be banned	1	0.5	8	4.3	113	61.1	63	34.1
E-cigarettes should carry warning labels about their potential risks, like other tobacco products do.	1	0.5	3	1.6	94	50.8	87	47.0
E-cigarettes should be regulated by the FDA for safety and quality standards	1	0.5	3	1.6	92	49.7	89	48.1
The sale of fruit or candy-flavored e-cigarettes should be banned.	2	1.0	38	20.5	92	49.7	53	28.7
Use of e-cigarettes indoors should not be allowed	1	0.5	9	4.9	97	52.4	78	42.2
There should be a minimum legal age to purchase e-cigarettes.	1	0.5	0	0	84	45.4	100	54.1

### Preparedness for e-cigarette use counseling

Among providers who were aware of e-cigarettes, the most frequently reported sources of information were from media or advertisement (83.8%) and patients (35.7%). Only 20.5% reported they have heard of e-cigarettes through professional sources (e.g. scientific literature). A minority of the respondents (27.6%) reported experience of caring for patients who had used e-cigarettes, and most respondents (84.3%) reported “somewhat” or “very” uncomfortable discussing e-cigarettes with the patients. In contrast, less than half of the respondents (46.5%) reported discomfort discussing smoking cessation treatment with the patients (Table 4).

**Table 4:** Counseling Preparedness on E-Cigarette Use among Participants.

	n	%
Information source (multiple choice, n = 184)		
Professional source (scientific literature)	38	20.5
Media or advertisement	155	83.8
Conversation with the patients.	66	35.7
Rarely heard of e-cigarette	18	9.7
Experience of caring for patients who used e-cigarette		
Yes	51	27.6
No	133	71.9
Comfort discussing with patients about e-cigarette		
Very uncomfortable	25	13.5
Uncomfortable	131	70.8
Comfortable	27	14.6
Very comfortable	1	0.5
Comfort discussing with patients about smoking cessation treatment		
Very uncomfortable	9	4.9
Uncomfortable	77	41.6
Comfortable	93	50.3
Very comfortable	5	2.7

One respondent did not provide answers to this set of questions, so the total does not equal 185.

## Discussion

To our knowledge, this study is the first to examine a sample of lung cancer specialists’ attitudes toward e-cigarettes and its regulation, an important emerging public health policy question. The professionals’ attitudes revealed from our results could be important for developing clinical guidelines and regulatory policy, addressing needs for informed, consistent advice for the patients and their families, and helping to shape social norms about e-cigarettes.

Most respondents believed that e-cigarettes are not safer than conventional tobacco cigarettes or smokeless tobacco, and feared that discussing e-cigarettes with the patients would encourage use. They did not consider it a smoking cessation treatment, and thus would not recommend them to smokers who do not want standard smoking cessation treatment or who failed to quit with conventional smoking cessation treatment. Although direct comparison with other studies is not possible due to differences in the sample characteristics, our results reflect a markedly more negative view of Korean lung cancer specialist physicians toward e-cigarette use. US primary care providers moderately agreed that e-cigarettes are safer than conventional tobacco cigarettes and smokeless tobacco, and few of them felt that discussing e-cigarettes with their patients would encourage its use [22]. More than half of the current smokers in the US (59.9%) believed e-cigarettes are less harmful than tobacco cigarettes [27].

Such strong negative views were not expected prior to the study, because we supposed that more knowledge about the scientific mechanism of e-cigarette would lead to lower risk perception and more positive views as an alternative to smoking. Higher education is associated with believing e-cigarettes were less harmful [6], and health professionals were somewhat positive toward e-cigarettes [22]. There could be several reasons for this finding. First, lung cancer specialist physicians routinely serve patients who got their cancer from smoking, and such experience could translate into more negative attitudes toward ‘smoking’, including e-cigarette smoking. They might fear that allowing e-cigarette use to their patients would make complete cessation more difficult, or lead to resumption of conventional tobacco cigarettes use, just as e-cigarette can be a gateway to regular smoking in the adolescent population. Second, media often covers injuries arising from e-cigarette-induced fires and health concerns from toxic chemicals in e-cigarettes in recent years [6], and this might affect their perception of the safety of e-cigarette. As respondents in our study answered that their source of information on e-cigarette was generally media and advertisement, it is likely that their perception was heavily influenced by such information about the negative aspects of e-cigarette.

Most respondents supported the full range of example e-cigarette regulations, including safety and quality checks, warning labels, advertisement ban, flavoring restrictions, minimum purchasing age, and restriction of indoor use. This might reflect the generally negative view of e-cigarette, as higher risk perception was associated with support for stricter regulation [27]. Currently, e-cigarette is mainly controlled by Tobacco Business Act by the Ministry of Strategy and Finance, and the Ministry of Drug and Food Safety in Korea does not strictly regulate e-cigarettes from the public health perspective. However, our respondents agreed that e-cigarette should be regulated in the same way as the conventional tobacco cigarettes product based on public health precautionary principles [29], and these views need to be considered in establishing public health policy.

Our finding that patients were an important information source about e-cigarettes for our respondents implies that this topic is already arising during usual conversations with the patients. To incorporate screening and counseling about e-cigarettes into their routine clinical assessment, they would need to have proper knowledge about pros and cons of e-cigarettes use and should be comfortable with discussing this topic with patients. However, most respondents learned about e-cigarettes from anecdotal information sources such as media and advertisements, or conversation with patients rather than through professional scientific resources, and reported low levels of comfort discussing e-cigarette with the patients. This is consistent with the findings with the US primary care providers [8,22]. Guidance from a professional society, such as recently published by the Tobacco Control and Smoking Cessation Committee of the International Association for the Study of Lung Cancer, would be helpful for clinicians to best advise patients about the safety and efficacy of e-cigarettes as a cessation tool [30].

Timely investigation of an emerging health issue, the use of a nationwide sample covering all geographic area, and the diversity of respondents across specialty and type of hospital are unique strengths of this study. However, generalizability to healthcare providers with other specialties will need to be established. In addition, although the survey items were developed mainly by adoption of items used in previous study, the questionnaire was neither guided by a theoretical framework and nor psychometrically validated. With the accumulation of our understanding of e-cigarette related health behaviors, future studies should examine this issue with more sound measurement tools.

Questions remain unanswered regarding the safety and effectiveness of e-cigarette as a smoking cessation aid, and whether and how it should be regulated. Provision of smoking cessation counseling and encouraging the use of FDA-approved medications should be the priority. However, given the increasing availability and use among cancer patients, families, and the public, lung cancer specialist physicians should guide their patient and families. Guidance from a professional society would be helpful for clinicians to be well prepared. In addition, regulatory guidelines that shape the social norm could protect people from the potential negative impact of free use of e-cigarettes. In our study, lung cancer specialist physicians in Korea doubted the safety of e-cigarette and use of e-cigarette as a smoking cessation aid, and supported stricter regulation. However, only 20% reported that they obtained information on e-cigarettes from the scientific literature, and many lacked adequate knowledge of scientific evidence. Nevertheless, the professionals’ view revealed from our study could help to develop clinical guidelines and regulatory guidance.

## Supporting information

S1 File Study Questionnaire in English and Korean. (DOCX)
