# Supplementary material for: Lung cancer specialist physicians’ attitudes towards e-cigarettes: A nationwide survey
Source: PLoS One. 2017 Feb 24;12(2):e0172568. doi: 10.1371/journal.pone.0172568 (PMC5325291; doi:10.1371/journal.pone.0172568)
Supplement: S1 File — (DOCX) [file pone.0172568.s001.docx]

**Study questionnaire in English**

1. Have you ever heard of electronic cigarettes before?

1) Yes 2) No

2. How harmful are electronic cigarettes compared to conventional tobacco cigarettes?

|  | Strongly disagree | Disagree | Agree | Strongly agree |
| --- | --- | --- | --- | --- |
| E-cigarettes are safer to use than conventional tobacco cigarettes. |  |  |  |  |
| E-cigarettes are safer to use than smokeless tobacco. |  |  |  |  |
| E-cigarettes could be a ’gateway’ to other tobacco use. |  |  |  |  |
| Discussing e-cigarettes with patients may encourage them to use e-cigarettes. |  |  |  |  |
| It is important to discuss e-cigarettes with the patients. |  |  |  |  |
| Smokers need to know about e-cigarettes. |  |  |  |  |
| E-cigarettes can be regarded as a type of smoking cessation treatment. |  |  |  |  |
| It is better to recommend e-cigarette to smokers who do not want to quit. |  |  |  |  |
| It is better to recommend e-cigarette to smokers who failed to quit with conventional smoking cessation treatment. |  |  |  |  |

3. How much do you agree with the following statements about the regulation of electronic cigarettes?

|  | Strongly disagree | Disagree | Agree | Strongly agree |
| --- | --- | --- | --- | --- |
| E-cigarette advertising should be banned |  |  |  |  |
| E-cigarettes should carry warning labels about their potential risks, like other tobacco products do. |  |  |  |  |
| E-cigarettes should be regulated by the FDA for safety and quality standards |  |  |  |  |
| The sale of fruit or candy-flavored e-cigarettes should be banned. |  |  |  |  |
| Use of e-cigarettes indoors should not be allowed. |  |  |  |  |
| There should be a minimum legal age to purchase e-cigarettes. |  |  |  |  |

4. Do you know that the Ministry of Food and Drug safety (Korea) does not currently regulate electronic cigarettes?

1) Yes 2) No

5. How did you get information about electronic cigarettes? (please select all)

1) I know and study the scientific literature.

2) I know through media reports.

3) I heard about electronic cigarettes from patients.

4) I have not heard much about electronic cigarettes.

6. Have you ever tried to treat patients who use electronic cigarettes?

1) Yes 2) No

7. How comfortable are you discussing with patients about electronic cigarettes?

1) Very uncomfortable 2) Uncomfortable

3) Comfortable 4) Very comfortable

8. How comfortable are you discussing with your patients about smoking cessation treatment?

1) Very uncomfortable 2) Uncomfortable

3) Comfortable 4) Very comfortable

9. Do you smoke now?

1) I smoke now

2) I smoked in the past but I quit

3) I have never smoked

10. What is your gender? 1) Male 2) Female

11. What year is your birth? ( )

12. What is your specialty?

1) Pulmonology 2) Thoracic Surgery

3) Hematooncology 4) Radiation Oncology

5) Other ( )

13. What is the year when you were board certified specialist? ( )

14. On average, how many clinical sessions do you see the patients per week?

( ) half-day sessions per week

16. On average, how many hours does a session last? ( ) hours

17. On average, how many patients do you see per session? ( ) patients per session

18. On average, how many lung cancer patients do you see per week? ( ) patients per week

19. What is the type of your institution?

1) University hospital (tertiary hospital)

2) Specialized cancer center

3) Secondary hospital

4) Other ( )

20. What is the type of your hospital?

1) Public

2) Private

3) Other (please specify: )

21. Where is your hospital office?

1) Major cities (metropolitan)

2) Small city

3) Rural area

22. What percentage of your patients are medicaid beneficiary? (Note: Medicaid program in Korea is government financial aid to persons who cannot afford national health insurance and represent 3% of population in Korea)

1) 0-5% 2) 5-10%

3) 10-20% 4) Above 20%

23. Did you drink in the last year?

1) I drink

2) I did, but stopped drinking

3) I have never drunk

**Study questionnaire in Korean**

1. 이전에 전자담배에 대해 들어보신 적이 있습니까?

1) 예 2) 아니오

2. 일반담배와 비교하여 전자담배의 위해 정도가 어떻다고 생각하십니까?

|  | 매우 동의함 | 동의함 | 동의하지 않음 | 전혀 동의하지 않음 |
| --- | --- | --- | --- | --- |
| 일반담배에 비해서 안전하다. |  |  |  |  |
| 무연담배(smokeless tobacco)에 비해 안전하다. |  |  |  |  |
| 다른 담배 사용의 관문(gateway)이 될 수 있다. |  |  |  |  |
| 전자담배에 대해 환자들과 논의를 하면 전자담배 사용을 부추길 수 있다. |  |  |  |  |
| 흡연자와 전자담배의 사용에 대해 논의하는 것은 중요하다. |  |  |  |  |
| 흡연자도 전자담배에 대해 알아야 한다. |  |  |  |  |
| 금연치료의 일종으로 생각할 수 있다. |  |  |  |  |
| 금연을 원하지 않는 흡연자에게 흡연 대신 권고하는 것이 좋다. |  |  |  |  |
| 통상적인 금연방법으로 실패한 흡연자에게 흡연 대신 권고하는 것이 좋다. |  |  |  |  |

3. 전자담배의 규제에 대한 아래와 같은 문장들에 대해서 얼마나 동의하십니까?

|  | 매우 동의함 | 동의함 | 동의하지 않음 | 전혀 동의하지 않음 |
| --- | --- | --- | --- | --- |
| 전자담배에 대한 광고는 규제되어야 한다 |  |  |  |  |
| 전자담배에 대해서도 일반담배와 마찬가지로 잠재적인 위험에 대해서 경고 문구가 들어가야 한다 |  |  |  |  |
| 전자담배도 안전성과 질에 대한 표준을 위해서 식약처로부터 규제를 받아야 한다고 생각하십니까? |  |  |  |  |
| 과일이나 사탕 맛이 나는 전자담배의 판매를 규제해야 한다 |  |  |  |  |
| 실내에서의 전자담배 흡연을 규제해야 한다 |  |  |  |  |
| 전자담배를 구매할 수 있는 법적 연령이 있어야 한다 |  |  |  |  |

4. 식약처에서 현재 전자담배에 대해 규제하고 있지 않고 있다는 사실을 알고 있습니까?

1) 예 2) 아니오

5. 전자담배에 대한 정보를 어떻게 접하셨습니까? (모두 골라주십시오)

1) 과학적 문헌을 고찰하여 알고 있다.

2) 언론 보도 등을 통해 알고 있다.

3) 전자담배에 대해 환자들로부터 들어보았다.

4) 전자담배에 대해 별로 들어본 적이 없다.

6. 선생님께서는 전자담배를 사용하는 환자를 치료해 본 경험이 있습니까?

1) 예 2) 아니오

7. 선생님께서는 전자담배에 관련하여 환자와 상담하는 것에 대해서 얼마나 편안하십니까?

1) 매우 편치 않다 2) 편치 않다

3) 편하다 4) 매우 편하다

8. 선생님께서는 금연치료에 관련하여 환자와 상담하는 것에 대해서 얼마나 편안하십니까?

1) 매우 편치 않다 2) 편치 않다

3) 편하다 4) 매우 편하다

9. 선생님의 흡연력은?

1) 현재 피움

2) 과거에는 피웠으나 끊었음

3) 한번도 피워본 적 없음

10. 성별은 어떻게 되십니까? 1) 남자 2) 여자

11. 출생년도는 어떻게 되십니까? ___________년

12. 전문과목은 무엇입니까?

1) 호흡기내과 2) 흉부외과

3) 혈액종양내과 4) 방사선 종양학과

5) 기타 ( )

13. 전문의 취득년도는 몇 년도 입니까? ______________년도

14. 평균적으로 주당 몇 세션을 진료하십니까? _________ 세션

15. 평균적으로 한 세션은 몇 시간 정도 입니까? _________ 시간

16. 한 세션당 진료하시는 환자는 몇 명 정도 입니까? 약 _________ 명

17. 1주일간 보시는 폐암 환자의 수는 평균 몇 명입니까? ( ) 명

18. 진료하시는 환자 중 폐암환자는 얼마나 차지하는 것 같습니까? 약 _________ %

19. 선생님의 근무기관의 형태는 무엇입니까?

1) 대학병원

2) 암전문병원

3) 이차병원

4) 기타 ( )

20. 선생님의 근무병원의 형태는 무엇입니까?

1) 공공의료기관

2) 민간의료기관

3) 기타 ( )

21. 선생님의 근무 병원은 어디에 소재하여 있습니까?

1) 대도시(특별시, 광역시)

2) 중소도시

3) 농어촌 지역

22. 선생님께서 보시는 환자 중 의료보호환자의 비율은 어느 정도나 되십니까?

1) 0-5% 2) 5-10%

3) 10-20% 4) 20%이상

23. 선생님께서는 최근 1년 기준으로 음주를 하십니까?

1) 음주 함

2) 음주하다 끊었음

3)원래 마시지 않음
